# Treatment of Industrial Brine Using a Poly (Vinylidene Fluoride) Membrane Modified with Carbon Nanotubes

**DOI:** 10.3390/membranes15080220

**Published:** 2025-07-23

**Authors:** Tshifhiwa T. Tshauambea, Soraya P. Malinga, Patrick G. Ndungu

**Affiliations:** 1 Department of Chemistry, Tshwane University of Technology, Private Bag X680, Pretoria 0001, South Africa; tshauambeatt@tut.ac.za; 2 Department of Chemistry, University of Pretoria, Private Bag X20, Hartfield 0028, South Africa; 3 Department of Chemical Sciences, University of Johannesburg, P.O. Box 17011, Doornfontein, Johannesburg 2028, South Africa; smalinga@uj.ac.za

**Keywords:** membrane technology, polyvinylidene fluoride, multiwalled carbon nanotubes, industrial brine

## Abstract

This study explores using polyvinylidene fluoride (PVDF) membranes modified with multi-walled carbon nanotubes (MWCNTs) to treat simulated and industrial brine from coal power stations. The MWCNTs were acid-treated and characterized using Fourier Transform Infrared Spectroscopy (FTIR), Raman, and nitrogen sorption at 77 K, Thermogravimetric analysis (TGA), and Transmission electron microscopy (TEM). The desired membranes were obtained by casting from a solution of N-Methyl-2-pyrrolidone, PVDF, various weight percentages of MWCNTs, and a small amount of polyvinylpyrrolidone. The acid treatment of the MWCNTs introduced oxygen moieties on the surface, and increased pore volume and surface area while maintaining crystallinity and structural integrity remain preserved. The maximum rejection rate achieved was 41.82% with 1 wt.% of acid-treated MWCNTs in the PVDF membrane. Acid-treated MWCNTs loaded membranes had an improved rejection rate, which was 5× higher than membranes without MWCNTs.

## 1. Introduction

One of the greatest difficulties facing the world in the twenty-first century is the provision of safe and affordable water. Furthermore, fast population increases, climate change, and water quality degradation continue to make it challenging for current water supplies to satisfy the ever-increasing demand for safe and affordable water [1,2]. Human activities, particularly industrialization, contribute to rising water shortages in emerging and developed countries by contaminating water resources and putting pressure on associated water resources [1]. People and industries rely on recycling water from different sources and the treatment and reuse of water for different purposes.

Wastewater from industries such as mining, oil and gas, coal-fired power plants, and pharmaceutical processing is difficult to treat in both developed and developing countries, including South Africa, due to its high total dissolved solids content and various pollutants [3,4]. This results in a relatively quick fouling of conventional membranes when using membrane processes and systems to treat such wastewater streams [5,6,7]. Brine, or the high concentration of salts found in industrial wastewater, has a persistent negative influence on the environment [8]. As a result, future policies and regulations may mandate that industrial wastewater have zero liquid discharge [8]. Salt composition in industrial brines differs depending on the water intake quality of the processes involved in the water treatment from which the final saline effluent is derived [9]. Currently, evaporation ponds are considered the most practical option for disposing of brine solutions from industrial activities, the ponds concentrate the brine, which leads to the salt precipitating into crystals, which are easy to remove from the ponds [9]. Evaporation ponds have disadvantages such as taking up useful land space, overflowing during heavy rain seasons, which leads to pollution in nearby waterbodies, and their functionality is highly dependent on climate conditions [9,10,11].

Membrane technology is one of several emerging technologies that can have a major impact on the sustainability and cost of wastewater treatment over conventional technologies [12]. This technology has advantages such as high removal capacity and flexible operational methods; it requires less energy and can be very cost-effective [13,14]. Membranes used in water treatment are commonly synthesized using organic (polymers) and inorganic (ceramics) materials [15,16,17]. In terms of comparison between organic and inorganic membranes, polymeric membranes are extensively utilized for water treatment because they have easy tuneable properties, improved processability, outstanding stability, high surface area for fast decontamination, selectivity to eliminate different pollutants, simple and require fewer steps during installation, and are more cost-effective than inorganic membranes [15,18]. Polymer membranes that have been demonstrated to be useful for water treatment include poly(vinylidene fluoride) (PVDF), polyethersulfone (PES), polyacrylonitrile (PAN), poly(vinyl alcohol) (PVA), poly(vinyl chloride) (PVC), polyethylene (PE), polypropylene (PP), polyamide, and chitosan [13,19]. Despite their outstanding stabilities and flexibility during installation, most polymer membranes are hydrophobic, which may lead to membrane fouling and poor selectivity [15,16,17,18]. Fouling results in low permeation flux, reduction or change in selectivity, and short membrane life spans [13,19,20,21].

To overcome membrane fouling and selectivity, fillers are introduced within the polymer used to make the membranes. Fillers are responsible for improving the mechanical strength, preventing swelling, and improving the chemical and thermal stability of polymeric membranes [13,19]. Nanofillers, when added to polymers within a range of 3–5 wt.% provide similar support properties to polymers that have been modified with 20–30 wt.% of micro-sized fillers. Thus, one of the key advantages nanomaterials have over micromaterials is a distinct weight advantage [19,20]. Nanoscale materials have become increasingly important as fillers due to their high specific surface area, which permits stronger interfacial interactions, in addition to their size [19,20].

Due to its distinct characteristics from its predecessors and their macro- and micro-sized counterparts, carbon nanotechnology has been the subject of extensive research in recent decades [13,20,22,23,24,25,26]. Among carbon nanomaterials, carbon nanotubes (CNTs) have drawn attention in the development of membranes and water treatment due to their inherent adsorption and sieving properties as well as their excellent mechanical and thermal stabilities [20,25,26,27,28]. Though they have excellent properties, CNTs are typically hydrophobic and tend to aggregate due to their strong intermolecular interactions, which include van der Waals forces and dipole–dipole interactions [23]. Aggregation properties of CNTs significantly reduces their dispersibility in a majority of solvents. In order to overcome this, CNTs can be functionalised using various chemical or physical techniques, leading to the introduction of functional groups on their surfaces [23,29,30]. The functional groups on CNT surfaces are essential for facilitating good dispersion in a polymer matrix, leading to improved membrane properties, including lower contact angles, enlarged pore size, increased rejection, anti-trade-off between permeability and selectivity, and antifouling properties [12,31,32].

Previous research on polymer membranes containing functionalized-CNTs (f-CNTs) indicated that these membranes could be highly effective in terms of permeability and salt rejection when used in desalination methods [25,26,27,28,33].

Sagar et al. synthesized f-CNTs immobilized membrane which was applied in DCMD desalination [25]. A maximum water flux of up to 36.8 kg/m^2^ and salt rejection of 99.9% were achieved using a feed of NaCl with a concentration of 10,000 ppm. An electron-spin nanofiber membrane was prepared by incorporating graphene oxide on polyvinylidene fluoride-co-hexafluoropropylene. Application of the membrane in desalination via air gap membrane distillation showed high flux with excellent salt rejection (100%) over 60-h operation using 3.5 wt.% NaCl solution [34].

The objective of this study was to explore and compare PVDF membranes modified with MWCNTs and f-MWCNTs and their application in treating real industrial brine solutions. Several researchers have studied the desalination of brine from simulated brackish and seawater [28,29,30,31]. Our focus in this investigation shifts towards the treatment of real industrial brine from a coal-fired power plant. This type of brine has varying compositions, higher levels of organic and inorganic pollutant concentrations, and increased variability. This study provides insights into the potential of polymer membranes to treat industrial brine wastewater with a mixture of inorganic and organic pollutants and to process water volumes with potentially lower energy usage and reduced pressure requirements.

## 2. Materials and Methods

No further modification and processing were done on all the reagents used. Multiwalled CNTs were bought from Timesnano, NMP solvent was purchased from Srichem, and diethyl ether was purchased from Merck. PVDF (Poly (vinylidene fluoride), with an average molecular weight of ~180,000 determined by gel permeation chromatography) and PVP (polyvinylpyrrolidone with an average molecular weight of 10,000) were purchased from Sigma Aldrich (Johannesburg, South Africa). Nitric acid (55%), sulfuric acid (98%), ethanol, and hydrochloric acid (32%) were purchased from accredited local suppliers. The industrial brine used in this work originated from a local coal-fired power plant (Eskom South Africa).

### 2.1. Methods

#### 2.1.1. Functionalization of Pristine-MWCNTs (p-MWCNTs)

MWCNTs were functionalized using methods adapted from Wand et al. [35]. In a round-bottom flask, 1.008 g of the purchased MWCNTs was mixed with 20 mL of HNO_3_ (55%) and 60 mL of H_2_SO_4_ (98%). The mixture was stirred for 90 min at 90 °C. The solution was cooled for a few minutes, and then diluted in 800 mL of deionized water and left standing for 12 h to allow separation to occur. The oxidized MWCNTs were washed several times with double-distilled water, filtered, and then dried in a vacuum oven overnight at 60 °C.

#### 2.1.2. Synthesis of Mixed Matrix Membranes with Carbon Nanomaterials

Membranes with different weight percentages (0.2%, 0.5%, 1%, and 2%) based on the weight of the polymer PVDF and of carbon nanomaterials (p-MWCNTs, and f-MWCNTs) were synthesized [24]. Firstly 0.08 g (0.5%) of p-MWCNTs was dissolved in 85 mL of NMP by sonication for 2 h. Then 14 g of PVDF followed by 1 g of PVP was added into the mixture, and the solution was stirred with an overhead stirrer for 24 h with the temperature kept at 60 °C. The prepared solution was removed from the stirring set-up, and it was left standing for another 24 h to remove all the air bubbles before casting it into a membrane. Before casting, the mixture was visibly inspected to ensure the CNTs had not settled out of the solution. To cast the membrane, the mixture was poured onto a glass plate and the casting knife set at 180 µm was used to cast the membrane. The glass plate was immersed in a water bath where the sheet peeled off into a membrane. The membrane was left in a water bath for 24 h to remove the residual solvent. After 24 h, the membranes were dried at room temperature.

### 2.2. Membrane Testing

For contact angle measurements, the analysis was done by dropping a few drops of ultrapure water using a needle at 5 different spots on each membrane (Sessile drop method). For the water intake capacity (*WIC*) measurement, the dry membranes were measured and thereafter the membranes were immersed in deionized water for 24 h and dapped with a paper towel before measuring the wet weight on the weighing balance. The following equation was used to calculate *WIC*%:

(1) $$WIC=\frac{W_{w}\;-\;W_{d}}{W_{w}}\;\times 100$$

where *W_w_* is wet weight, *W_d_* is dry weight.

Each membrane sample (0.00126 m^2^) was compacted using deionized water for an hour at 200 kPa until a stable flux was obtained. This was done before taking measurements for both water flux and solute flux. The water flux and solute flux were calculated using Equation (2) where *J_w_* is replaced by *J_s_* for solute flux.

(2) $${J_{s}/J}_{w}=\frac{Q}{A\cdot \Delta t}$$

where:

*J_w_* is the water flux (L/m^2^∙h)

*J_s_* is the solute flux (L/m^2^∙h)

*Q* is the volume of the permeated water (L)

*A* is the effective area of the membrane (m^2^)

Δ*t* is the permeation time (h)

Synthesized membranes were further tested if they were influential in removing salts from industrial brine and simulated brine. The simulated brine solution was prepared by mixing Calcium (Ca) = 601.31 mg/L, sodium (Na) = 3028.99 mg/L, Magnesium (Mg) = 102.13 mg/L, and chlorine (Cl) = 1584 mg/L (prepared from calcium chloride, magnesium chloride and sodium chloride) with the concentration of ions chosen based on the concentration before on real industrial brine solution [9].

#### Brine Testing

The rejection tests were done in terms of the removal of salts from the brine and the removal of total organic carbons using a TOC analyser. There was no pre-treatment of the industrial brine before analysis. A conductivity meter was used to measure conductivity in mS and TDS in mg/L. The conductivity and TDS of the simulated brine and industrial brine were measured before treating (feed concentration) both the solution with the membranes and after treatment (permeate concentration) [36,37,38]. The rejection of salts was calculated using,

(3) R%=1−CpCf×100

where:

*R* is rejection (%)

*C_p_* is the permeate concentration (mg/L) measured TDS.

*C_f_* is the feed concentration (mg/L) measured TDS.

Membrane samples, with each loading, were synthesized in duplicate, and each sample was tested.

The studies on the removal of total organic carbons were done only on the industrial brine.

## 3. Results and Discussion

### 3.1. Characterization of Nanomaterials

#### 3.1.1. FTIR Analysis

**Figure 1:**
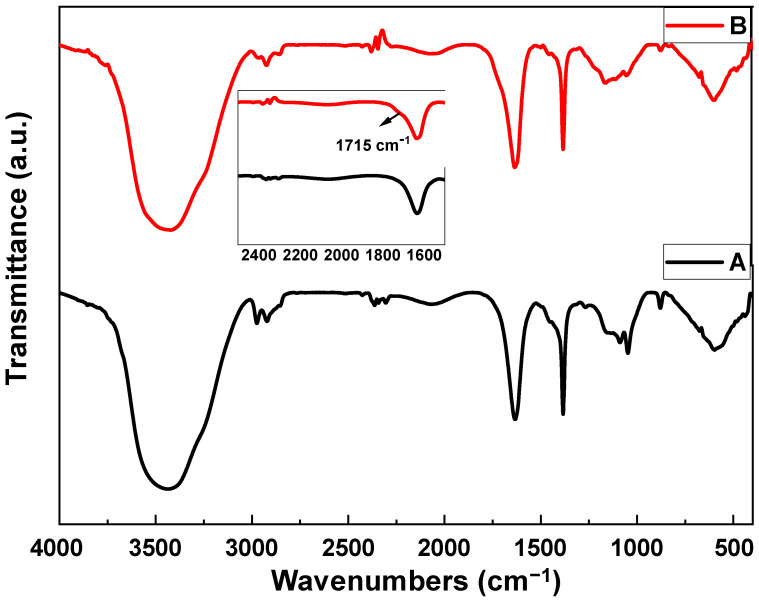
FTIR spectra of (A) MWCNTs and (B) f-MWCNTs.

Figure 1 shows the FTIR spectra of MWCNTs and f-MWCNTs, which provides some insight on the functional groups on the surfaces of the MWCNTs, as well as certain modifications after treating the MWCNTs samples with strong acids (H_2_SO_4_ and HNO_3_) at 90 °C. The FTIR spectra of MWCNTs and f-MWCNTs show similarities in terms of functional groups identified, though, from the MWCNTs spectra, the intensity of the C-H at 1383 cm^−1^ is higher than that of the f-MWCNTs and this may indicate additional functional groups on the f-MWCNTs. Both the MWCNTs and f-MWCNTs have the C=C bond (stretch) at around 1630 cm^−1^, and only the f-MWCNTs samples had a small shoulder peak at 1715 cm^−1^ caused by vibrations of C=O bonds. The appearance of the C=O vibrations on the f-MWCNTs indicate the acid treatment successfully introduced new surface groups to the carbon nanomaterials. The symmetric and asymmetric stretching vibrations of the methylene group are assigned to the peaks observed at 2975 cm^−1^ and 2921 cm^−1^ on the MWCNTs. On the spectrum of the f-MWCNTs, the methylene group peaks appear to have shifted to 2966 cm^−1^ and 2925 cm^−1^ with less intensity. The C-OH stretching vibration is assigned to the 3445 cm^−1^ for MWCNTs and the 3427 cm^−1^ for the f-MWCNTs. The shoulder peak (see insert on Figure 1) on the f-MWCNTs and peak shifts accompanied by lowering of intensities gives some idea that the addition of oxygen functional groups has occurred on the surface [39,40]. The presence of hydrophilic oxygen functional groups in membrane synthesis speeds up the solvent-non-solvent exchange process [41,42]. This results in membranes with large pores, improving the water flux even at low operating pressures [43].

#### 3.1.2. XRD Results

**Figure 2:**
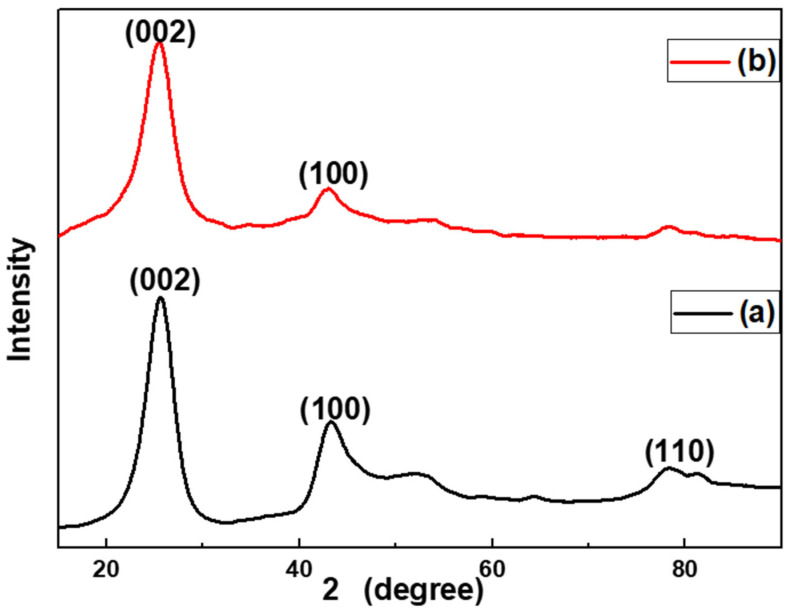
XRD patterns of (a) MWCNTs and (b) f-MWCNTs.

The XRD patterns of MWCNTs show diffraction peaks at 2θ = 25.52°, 43.77°, and 78° corresponding to (002), (100), and (110) reflection planes (Figure 2), and these are patterns typically associated with graphitic carbon. The f-MWCNTs have two diffraction peaks at 2θ = 25.31° and 43.01°, and a relatively low intensity and broad peak around the (110) reflections plane. The (002) diffraction peak can be used to provide some information about the crystalline structure of CNTs. The increase in the FWHM and the peak shift of the (002) diffraction peak from 25.52° to 25.31° (see Table 1) indicates a decrease in the crystallinity of the MWCNTs after acid treatment (002) [44]. The 90-min acid treatment used in this study indicates samples were functionalized with minimal degradation of the crystallinity of the MWCNTs [45,46]. The preserved crystallinity is beneficial for interaction at polymer/f-MWCNTs interfaces, which may enhance the mechanical strength of membranes modified with MWCNTs [47].

Further investigation using Bragg’s law (nλ = 2dsinθ) shows that there is an increase in the interspacing distance from 0.3486 nm to 0.3515 nm for f-MWCNTs. Although the values are close, the small difference can be used as an indication of some degree of functionalization, and this can be attributed to the addition of oxygen functional groups (as per FTIR findings).

#### 3.1.3. Raman Analysis

**Figure 3:**
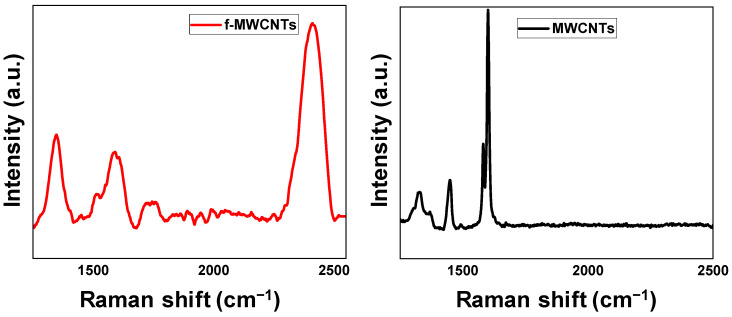
Raman spectrum of MWCNTs and f-MWCNTs showing their D and G bands.

The results of the Raman analysis are presented in Figure 3. From Figure 3, the MWCNTs exhibited peaks with the D band at 1449 cm^−1^, and the G band at 1601 cm^−1^ Similarly, the f-MWCNTs exhibited peaks with the introduction of defects during oxidation resulting in the shifting of the peaks [48], thus the D band appears at 1347 cm^−1^, the G band at 1597 cm^−1^, and 2D at 2414 cm^−1^, the overtones appears at 1515 and 1738 cm^−1^ respectively. The D band is due to double resonance scattering caused by the structural defects during the attachment of different chemical groups on f-MWCNTs and is typically attributed to sp^3^ carbon [45,49]. The vibration of the graphitic carbon (sp^2^) along the nanotube axis is responsible for the G band [50,51]. The functionalization of MWCNTs results in changes in the intensity and the increase of the FWHM of G and D bands, as well as shifts in the peak positions of the G and the D bands [52,53]. Intensified structural defects caused by strong acid oxidation result in shifts in the position of the Raman peaks [54] and a decrease in the intensity of the G and D bands, the results obtained here indicate that MWCNTs underwent acid oxidation with minimum structural defects. The G′ on the f-MWCNTs is the result of two phonons scattering around the Brillouin zone’s K point [55]; this band is the defect-induced D-band’s second overtone [56]. The results from the Raman analysis correlate with the FTIR observations; specifically, the increase in the FWHM for both the D and G bands indicating increased defect structures due to the modification, and the presence of new functional groups (FTIR analysis) on the surfaces of the f-MWCNTs. The crystallinity of f-MWCNTs is confirmed by the G band which represents the graphitic structure of carbon (sp^2^), this is in line with the XRD results.

The introduction of defects, via chemical oxidation in this case, on the MWCNTs surfaces can be inferred from the I_D_/I_G_ ratio. Results in Table 2 show that the I_D_/I_G_ ratio is higher for the f-MWCNTs (0.85) than the I_D_/I_G_ of the MWCNTs (0.26). This corresponds with the addition of the oxygen-containing functional groups on the MWCNTs surface, as seen in FTIR results. The increase in I_D_/I_G_ value for f-MWCNTs not only confirms the defects on the carbon surface, but also some change in the degree of graphitization [48]. The defects on the carbon surfaces and introduction of new chemical functional groups are crucial in membrane applications, as they improve the dispersal properties of the CNTs in the polymer matrix, promote hydrophilicity, and play a role in the reduction of membrane fouling [57].

The G and 2D prime band of f-MWCNTs shows a clear separation and this characteristic has been seen in other CNTs where the 2D has been associated with the overtone mode of the D band [58,59]. The increase in the FWHM on the f-MWCNTs is attributed to the oxidation process at higher temperatures for a relatively long period of time. The results obtained here are comparable to what has been reported in other studies where the oxidation of the MWCNTs is confirmed by the shifting of the D and G bands [48,60].

#### 3.1.4. Thermal Stability (TGA Analysis)

**Figure 4:**
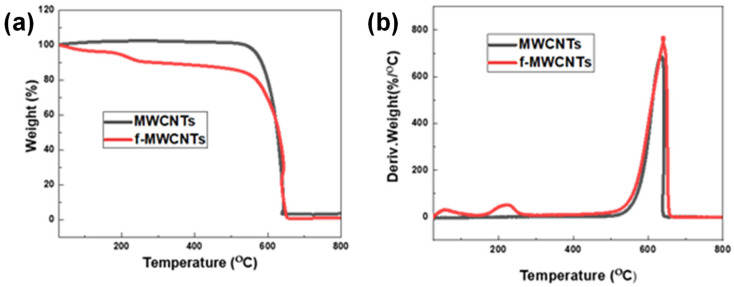
Thermal stabilities of MWCNTs and f-MWCNTs as represented by their (**a**) TGA curves and (**b**) derivative weight loss curves.

Thermal stability studies, presented in Figure 4, show that MWCNTs are stable, with decomposition starting at a temperature of 600 °C, which is similar to previous reports [48,61]. The f-MWCNTs, on the other hand, decompose in three steps, with the first step starting at 40 °C and reaching a maximum of 56 °C representing the removal of water molecules adsorbed by the samples [62], this accounts for the loss of weight of 3.6%. The second decomposition, which accounts for the weight loss of 8.31%, is caused by the loss of the hydroxyl and carbonyl groups attached to f-MWCNTs surface starting at 180 °C and reaching maximum decomposition at 218 °C [48]. The loss of weight of 8.31% on the f-MWCNTs is a further indication of the functionalization of the MWCNTs with various oxygen moieties attached to the surface, which corroborates the FTIR and Raman results. The last step of decomposition is at 620 °C, which is due to the oxidation of the remaining carbon frameworks [63].

The FTIR spectrum of f-MWCNTs did provide some indication that the treatment had introduced various oxygen functional groups. Further analysis with XRD and Raman indicated and supported the FTIR analysis in terms of the functionalization of the f-MWCNTs. The TGA decomposition peak at 218 °C on the f-MWCNTs, and the lack of a similar peak on the MWCNTs further confirms that there are various oxygen moieties on the f-MWCNTs surface. TGA provided insight into the thermal stability of the nanotubes before and after functionalization. The 8.3% weight (%) loss observed with the f-MWCNTs is a semi-quantitative measure of the degree of functionalization of the nanotubes. In addition, the derivative weight loss peak for the MWCNTs and f-MWCNTs are similar, which shows the oxidative treatment was not only successful, but it did not significantly damage or degrade the nanotube properties. From the results obtained, the f-MWCNTs are likely to disperse and incorporate into a polymer matrix and the resulting membranes will likely have improved physical properties due to the addition of the nanotubes.

#### 3.1.5. TEM Analysis

**Figure 5:**
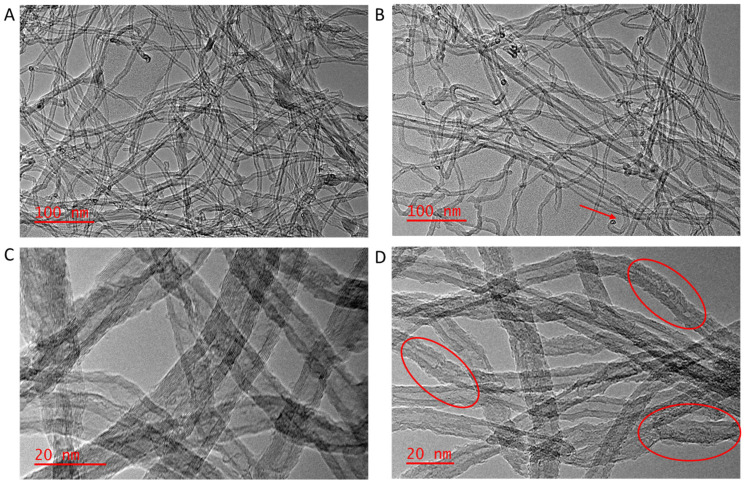
TEM images of (**A**) MWCNTs, (**B**) f-MWCNTs, (**C**) higher magnification image of MWCNTs, and (**D**) higher magnification image of f-MWCNTs. Arrow on micrograph (**B**) indicates nanotube end, and circles in (**D**) highlight walls of the nanotubes.

Transmission electron microscopy (TEM) analysis of the MWCNTs and the f-MWCNTs are presented in Figure 5. Figure 5 shows some minor damage to the wall structure of the MWCNTs. Most of the outer and some of the inner walls of the f-MWCNTs were distorted, with some indication of a change in the roughness of the wall surface (highlighted in red circles Figure 5D). Figure 5A shows MWCNTs that are highly entangled, caused by the strong van der Waals forces [57]. The acid treatment at high temperature led to the cleavage of some nanotubes, opening of nanotubes caps, and ingress of the treatment into the inside pores as evidenced by roughening of the inner walls (Figure 5B).

The changes on the surface of the f-MWCNTs show that oxidation under acidic conditions does not only bring chemical change but also affects the morphology, which is an indication of the disordered carbon on the surface of MWCNTs. The I_D_/I_G_ ratio from Raman increased from MWCNTs to f-MWCNTs. This agrees with defects in the outer and inner walls of f-MWCNTs as seen on TEM images. This characteristic is an indication of disordered carbon on the surface. Similar characteristics to what was observed in this work were reported where small damages on the surface of the MWCNTs before acid treatment were observed and this is associated to the purification by the manufacturer to remove amorphous carbon and catalytic metal [64]. While the defects on the surface of the nanotubes may be detrimental to the mechanical properties of the CNTs, they are beneficial in terms of the dispersion properties of the nanotubes, which facilitates the interfacial bonding with the polymer matrix. Also, the defect sites may also contain chemical moieties that enhance ion-exchange processes or absorption and rejection mechanisms, resulting in the overall improvement of the membrane performance.

The method of functionalization has a significant impact on the degree of surface functionalization of the nanotubes. A study done on the duration of the treatment of the MWCNTs revealed that the weight percent of oxygen functional groups on MWCNTs increases with increasing oxidation time [46,48]. This was achieved when medium-concentration mixtures of HNO_3_/H_2_SO_4_ were used, as reflux at high concentrations may result in the structural damage of the MWCNTs [46,64]. In contrast, the 90-min reflux time used in this study, with concentrated acid mixtures did result in alteration of the surface chemistry of the samples as well as the morphology, but the thermal stability remained similar to the untreated samples.

#### 3.1.6. Textural Characteristics of the MWCNTs

The surface area of MWCNTs is highly affected by modifications such as surface functionalization. The theoretical studies reported that the surface area of CNTs as a function of the number of walls and diameters is in the range between 50–1315 m^2^/g, as reported by Piegney et al. [65]. The experimental surface area of MWCNTs ranges between 10–50 m^2^/g, and it is mostly the function of several walls. From Table 3, the surface area of f-MWCNT is 244.5 m^2^/g which is an increase from 202.3 m^2^/g of MWCNTs.

**Figure 6:**
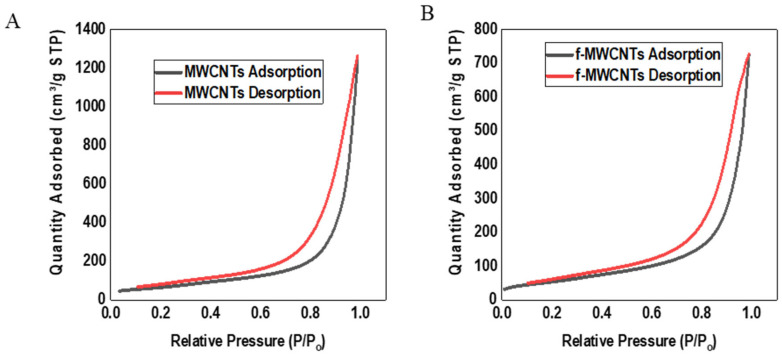
Nitrogen adsorption isotherms of (**A**) MWCNTs and (**B**) f-MWCNTs.

It is expected that functionalizing MWCNTs results in higher surface area, due to such factors as roughening of the walls of the nanotubes (see Figure 5B), opening the ends of the nanotubes, or detangling the CNTs. The BET theory assumes uniform adsorption over the surface, whereas the adsorption sites on MWCNTs differ due to aggregation and defects, as demonstrated in TEM pictures of f-MWCNTs [65]. The BET surface area of f-MWCNTs (244.51 m^2^/g) reported in this work is similar to the 244.86 m^2^/g (MWNTs) which was reported by Shabaan et al. [66]. The increase in the BET surface area of f-MWCNTs confirms that acid treatment not only added oxygen functional groups successfully on the surface, but also detangled the nanotubes, roughened the walls of the CNTs, and opened up some ends of the MWCNTs [67]. The sorption isotherms of both MWCNTs and f-MWCNTs display a type IV isotherm with a hysteresis loop, which is a feature of a mesoporous material [68,69,70,71,72], as shown in Figure 6.

Functionalization of MWCNTs under acidic medium resulted in an increase in the pore volume and a similar observation was seen when MWCNT-COOH functionalised using 3:1 H_2_SO_4_ and HNO_3_ had a total pore volume of 1.410 cm^3^/g as compared to the 0.570 cm^3^/g of the MWCNTs [73].

### 3.2. Membrane Characterizations

#### 3.2.1. ATR-FTIR

**Figure 7:**
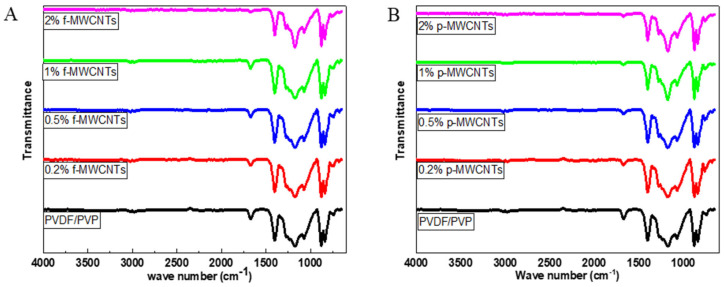
ATR-FTIR spectrum of PVDF/PVP membranes loaded with different wt.% of (**A**) f-MWCNTs and (**B**) p-MWCNTs.

The FTIR spectra of the PVDF/PVP membrane and membranes doped with varying wt.% of p-MWCNTs, and f-MWCNTs were recorded using an ATR accessory, and all the membranes had similar characteristics peaks (Figure 7). Peaks at 1402 cm^−1^ and 1173 cm^−1^ are due to C-C’s stretching and deformation vibrations on the aromatic ring and C-F from the PVDF [74]. The PVP main characteristics peaks are the 1674 cm^−1^ and 1272 cm^−1^ due to the stretching and bending vibrations of the C=O and C-N [24,75,76,77]. Weak peaks at 2977 and 3021cm^−1^ are because of the stretching vibrations of C-H. The peak arising from 3020 cm^−1^ and 2971 cm^−1^ is caused by stretching vibrations from the CH_2_ from polymer PVDF [74,78].

The relatively low weight percentages of the MWCNTs and f-MWCNTs in the polymer membrane did not result in the observation of any key peaks from the nanomaterials within the nanocomposites. In addition, some of the key peaks occur in the same region as the polymer peaks and could have been obscured by the more intense polymer vibrational peaks.

#### 3.2.2. XRD Analysis

**Figure 8:**
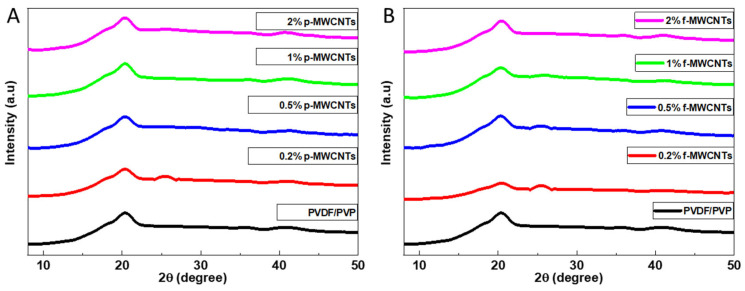
X-ray diffraction patterns of synthesized membranes loaded with 0.2–2% of the (**A**) f-MWCNTs and (**B**) p-MWCNTs.

The crystallinity of nanocomposite membranes was evaluated by making use of XRD. All the synthesized membranes in Figure 8 show a sharp peak corresponding to the (110) diffraction peak. The (110) diffraction peak appears at 2θ region of 20.28–20.43° respectively. In prior studies, it was discovered that β-phase of PVDF occurs at 2θ region of 20.2–20.6° [79]. The β-phase in this study was caused by stretching, elongation forces, and pulling during the casting of the membrane [80]. A weak peak (211) diffraction plane is observed at 2θ = 40.1° on the bare PVDF/PVP membrane. This peak has previously been related to the γ-phase of the PVDF [79]. From Figure 8 0.2 and 0.5% f-MWCNTs shows a diffraction peak at 2θ = 25° and this peak is due to the f-MWCNTs as it was seen in the previously on X-ray diffraction pattern of f-MWCNTs. The outcomes acquired in this investigation exhibit similarity to prior observations documented in the literature, wherein the β-phase of PVDF/P[MMA-IL]-MWCNTs manifested at 2θ = 20.6°. In the present study, the β-phase is discerned at 2θ = 20.2°, aligning with the established findings in the field [81].

#### 3.2.3. SEM (Surface and Cross-Sectional)

Studies and comparisons of the membrane surface morphology were done using SEM. The diameter of the membrane pores was measured using ImageJ (version 1.53v), the image of the membrane captured from SEM was loaded on ImageJ and the scale was set to 10 μm (from the scale on the image), for each membrane 200 pores were measured by drawing a line across each pore. A distribution curve was plotted using the Origin software program, and each membrane’s average diameter was obtained. The diameter measurements of the pores are shown by their distribution curves underneath each membrane’s SEM image.

**Figure 9:**
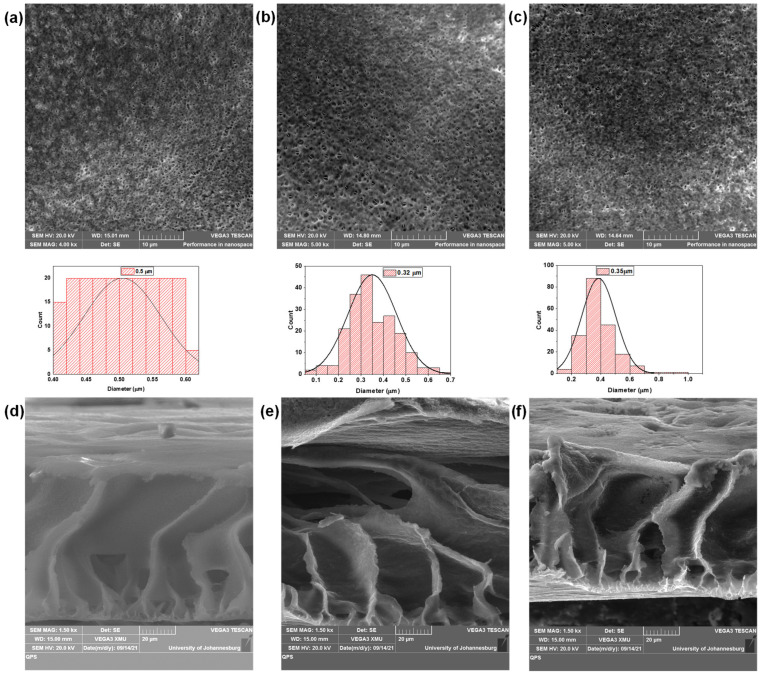
SEM images showing surface of (**a**) PVDF/PVP with corresponding pore size distribution below the micrograph, (**b**) PVDF/PVP/ 1 wt.% MWCNTs with corresponding pore size distribution below the micrograph, and (**c**) PVDF/PVP/ 1 wt.% f-MWCNTs with corresponding pore size distribution below the micrograph, and the cross-section micrographs of (**d**) PVDF/PVP, (**e**) PVDF/PVP/ 1wt %p-MWCNTs, and (**f**) PVDF/PVP/ 1 wt.% f-MWCNTs.

From Figure 9, all of the synthesized membranes generally exhibited a relatively large number of pores on the membrane surface, and incorporation of the MWCNTs and f-MWCNTs had an influence on the overall pore size distribution. The PVDF/PVP membrane pore distribution curve was centred at 0.5 µm with similar count for the various pore sizes. The 1 wt.% PVDF/PVP/MWCNTs pore size distribution curve was centred at 0.32 µm with a higher count for the smaller pore sizes. The 1 wt.% PVDF/PVP/f-MWCNT’s pore size distribution curve was centred at 0.35 µm with counts distributed over a narrower range over smaller diameters. Addition of the MWCNT’s or f-MWCNT’s resulted in a higher count of pores with smaller diameters, indicating the nanotubes do affect the formation of the surface pores. PVP is well known as a pore former in PVDF membranes, and it’s addition to the casting solution enhances the kinetics and phase separation processes during casting and results in greater number of pores [82,83], and in our system wide distribution of large pores on the surface. The addition of the nanotubes affected the pore formation process, possibly due to some favourable interactions between the PVP and the nanotubes, and resulted in smaller pores and narrower distributions. Our results are similar to other reports in the literature, were carbon nanotubes have been observed to result in the formation of smaller surface pores [47]. Prior reports in the literature have attributed the fast exchange between solvent and non-solvent during the phase inversion process to the hydrophilic groups on the surface of the nanotubes [84,85,86]. The cross-sectional image of the PVDF/PVP membrane (Figure 9d) showed the expected asymmetric morphology and structure with finger-like macro-voids [87,88]. The addition of the nanotubes had a minimal effect on the formation of the macro-void structures; however, the root-like structures at the bottom of the membrane observed with the PVDF/PVP membranes were not prominent on the PVDF/PVP/ 1 wt.% MWCNTs and the PVDF/PVP/ 1 wt.% f-MWCNTs.

#### 3.2.4. Contact Angle and Water Intake Measurements

Results of the WIC % and contact angle measurements (an average from measurements of five different spots on each membrane) are represented in Table 4.

The membranes with smaller contact angles (<90°) resemble a more hydrophilic nature than membranes with higher contact angles (>90°) [89,90]. The bare membrane (PVDF/PVP) has a contact angle of 85.45°, which is not much larger than the value of 83.8° reported by Guangyong et al. This high contact angle is related to the intrinsic hydrophobic nature of PVDF [91]. Recent studies show that PVDF without the modification by PVP has a contact angle of 141°; when comparing it to the contact angle of 85.45° obtained in this work, it can be confirmed that adding PVP improves the hydrophilicity of the PVDF [92].

Incorporating the carbon nanomaterials in the membranes further enhances the membrane’s hydrophilicity, and hydrophilicity increases with increasing the wt.% of nanomaterials. As the membranes’ hydrophilicity and pore size improve, their water intake capacity (WIC) % increases. Previously, enhanced hydrophilicity was linked to nanoparticle migration towards the membrane/water interface, resulting in a decrease in interface energy during the phase inversion process [93]. From Table 4 the contact angle membranes decrease in the following order: MWCNTs 79.41–72.63° > f-MWCNTs 72.57–68.25°. This order shows that the membranes with nanomaterials rich in oxygen functional groups are more hydrophilic than those with fewer oxygen functional groups. From the results obtained in this study, it can be confirmed that the hydrophilicity of a membrane varies according to the chemical composition of the additive, which in this study are PVP, MWCNTs, and f-MWCNTs [94]. The improved hydrophilicity enables the formation of a hydrated layer, thereby enhancing the water intake capacity of the membranes, which has been reported in previous studies, and shown to improve the water flux and anti-fouling performance [95,96].

### 3.3. Performance Tests on the Membranes

#### 3.3.1. Water Flux and Permeate Flux Tests

**Figure 10:**
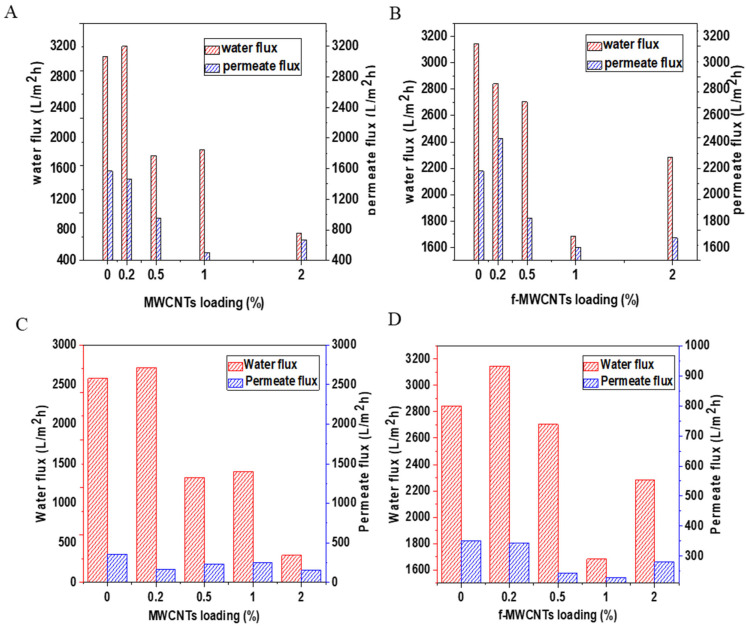
Water and permeate flux of PVDF/PVP membranes loaded with (**A**) p-MWCNTs and (**B**) f-MWCNTs (simulated brines) and (**C**) p-MWCNTs and (**D**) f-MWCNTs (industrial brines).

Water and permeate flux were done at a pressure of 100 kPa and time t = 10 min. As seen from Figure 10, the bare membrane (PVDF/PVP) water flux was increased upon the addition of 0.2 wt.% p-MWCNT nanomaterial; this is due to the water transport promoted by the nano-corridors, which forms when polymers interact with the nanomaterial’s functional groups during phase inversion process [97]. Previous studies have shown that increasing nanomaterial loading in the polymer matrix may result in agglomeration of nanomaterials, causing pore blockage, and this may be the reason for the decrease in water flux with increasing nanomaterial loading [98]. The pure water flux of the membranes decreased when wt.% of the nanomaterials was increased from 0.5 wt.%, increasing the nanomaterials in the casting solution results in blocking the pore connection, and this hinders water transportation [99,100].

**Figure 11:**
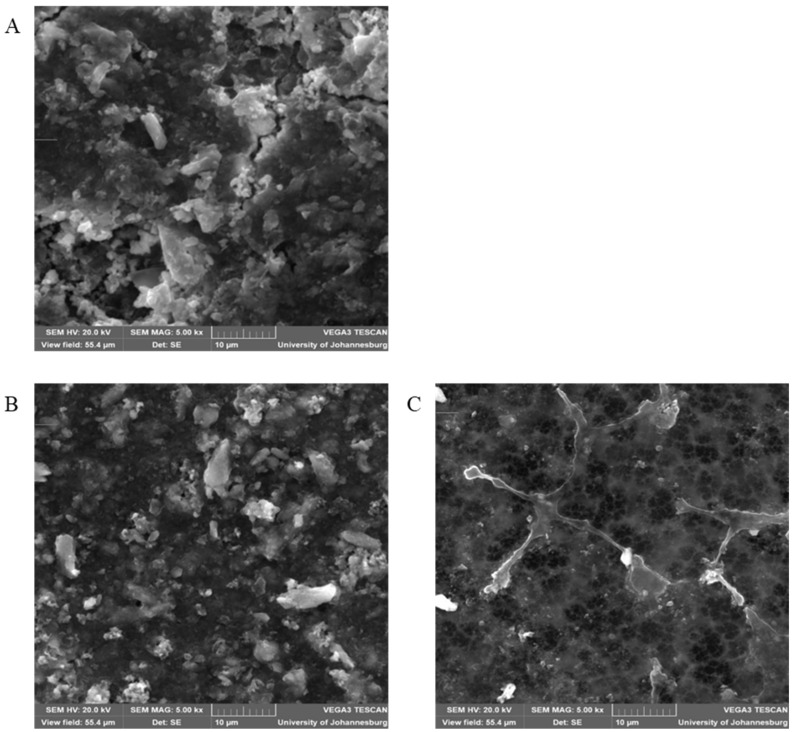
Shows accumulation of solutes and particles on membrane surfaces of (**A**) PVDF/PVP, (**B**) PVDF/PVP/1 wt % p-MWCNTs, and (**C**) PVDF/PVP/ 1 wt % f-MWCNTs.

The permeation flux tests were done using the industrial brine solution as well as simulated brine solution, and the results from Figure 10 shows that all the synthesized membranes have permeate flux which is much lower than the water flux. A decrease in the membrane permeate flux indicates fouling that occurred on the surface of the membrane. The SEM images of the membranes obtained after treatment of the brine solution (Figure 11) show that each membrane has a layer deposited with crystals and particles blocking pores on the membrane surface. The decrease in the permeate flux of the simulated brine is caused by friction between the membrane matrix with friction between water and ions as it controls water flow through the membrane [101,102]. The large decrease in the MWCNTs loaded membrane is associated with the smaller pores as was seen previously on the SEM images showing the smaller diameter of the membrane pores.

#### 3.3.2. Rejection Tests: Effects of Nanomaterial Loading

**Figure 12:**
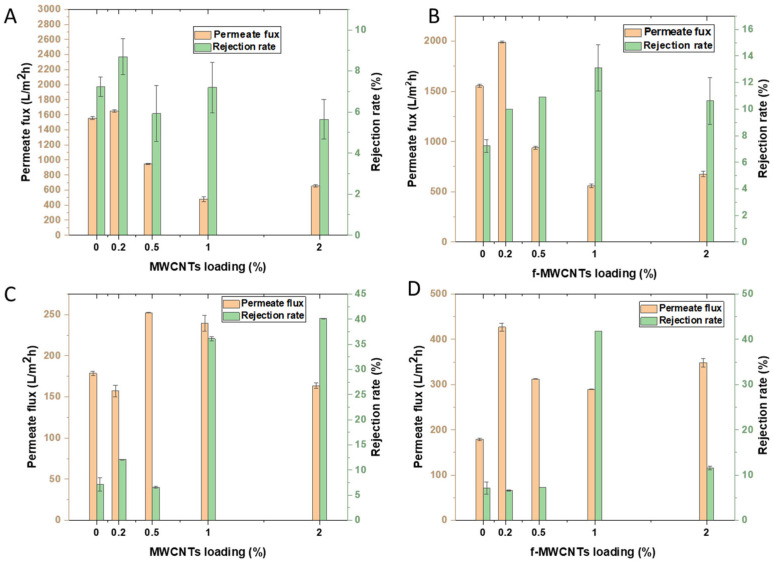
Effects of nanomaterials loading on the PVDF/PVP membranes (**A**) simulated brine on MWCNTs loaded membranes, (**B**) simulated brine of f-MWCNTs loaded membranes, (**C**) industrial brine on MWCNTs loaded membranes, and (**D**) industrial brine on f-MWCNTs loaded membranes.

The effects of nanomaterials dosage were done to determine the optimum wt.% loading of the nanomaterials (MWCNTs, and f-MWCNTs) that resulted in good membrane performance. The membranes without MWCNTs had a rejection rate of 7.23% when using simulated brine. With the addition of 0.2 wt.% loading of MWCNTs, the rejection rate increased to 8.69%. After adding 0.5 wt.% of MWCNTs to the membrane, the simulated brine rejection rate dropped to 5.93%, which is related to poor distribution of the nanotubes in the polymer matrix. However, the rejection rate increased to 7.33% with the 1 wt.% loaded membrane. Another decline in the rejection rate to 5.65% was observed, after loading the membranes with 2 wt.% of MWCNTs, and is likely associated with the agglomeration of the MWCNTs resulting in defects in the polymer matrix, which may affect the membranes performance [92]. The performance of the MWCNTs loaded membranes on industrial brines yielded results that are different from those of the simulated brine with a rejection rate up to 40.13%. The bare membrane has a low rejection rate at 7.15%, which is similar to the simulated brine. When the membrane was loaded with 0.2 wt.% MWCNTs, the rejection rate increased to 12.08%, but dropped to 6.1% with 0.5 wt.% loading, and then further increases were observed with the 1 (38.1 5 rejection rate) and 2 wt.% (40.1% rejection rate) MWCNTs loaded membranes. The membranes with f-MWCNTs incorporated in their matrix have a slightly higher rejection rate on simulated brine compared to the rejection rate of membranes with MWCNTs. The rejection rate increased from 9.99% when 0.2 wt.% of f-MWCNTs was introduced and increased at 0.5 wt.% to 10.92%, further increased to 14% at 1 wt.%, and then decreased to 10.61% at 2 wt.%. For industrial brine, the f-MWCNTs membrane loaded membranes showed rejection rates of 7, 5, 6, 44, and 12% for the 0, 0.2, 0.5, 1, and 2 wt.% loaded membranes. From Figure 12, the membranes loaded with 1 wt.% of the f-MWCNTs performed better than the other membrane loadings for both the simulated and industrial brines. The 1 wt.% loading may have resulted in enough oxygen moieties required in the membrane matrix for ion exchange, another factor could be the homogenous distribution during the preparation of the casting solution [103]. Increasing the wt.% to 2 resulted in a decrease in the rejection rate with both simulated and industrial brines, this may be due to the poor distribution of the high-content nanomaterials in the casting solution [104]. Overall, the 1 wt.% f-MWCNTs membrane has a higher rejection rate than the MWCNTs, and this confirms that introducing functional groups on MWCNTs assists in opening pores at the tips leading to access of the walls inside the nanotubes, introducing charged oxygen containing groups on the surface of the CNTs which assist with the rejection of ions in the brine [104]. Comparing permeate flux and rejection rates, it can be seen that membranes with low permeate flux have higher rejection rates when compared to membranes with high permeate flux. The low permeate flux is an added advantage as it permits more interaction between the contaminants and the nanomaterials within the membrane’s matrix.

The overall rejection obtained in this work was ~41.8%, which is low in comparison to the rejection rates that have been reported in the literature. This is due to the large pore size on the membrane and the use of simulated and real industrial brine solutions, containing multiple salts at high concentration. In contrast, the membranes reported in Table 5 were tested on synthetic brine containing a single salt, which may result in higher rejections under more controlled conditions. Moreover, the membranes synthesized in this study exhibited high water and permeate flux at a low operating pressure of 1 bar, whereas the membranes reported in Table 5 required significantly higher pressures (10–16 bar) to achieve comparatively lower flux values. The use of low pressure (100 kPa) makes the membrane synthesized in this study essential for saving energy and may be applicable in desalinating larger volumes of wastewater [105]. Rejection rates of up to 35% of 0.06 M NaCl have been reported before, where the temperature-induced phase separation technique was used to synthesize the PVDF-CNT [105].

#### 3.3.3. Effects of Contact Time

**Figure 13:**
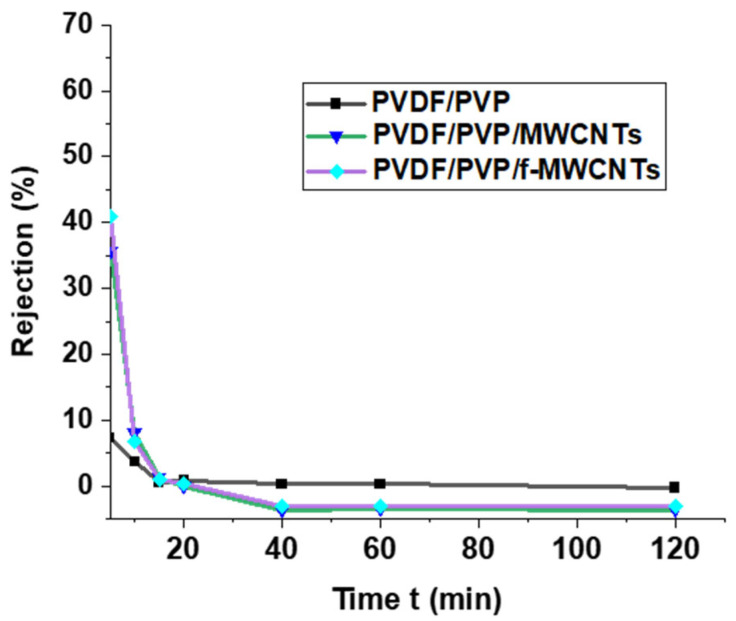
Effects of contact time on the removal of salts from industrial brine.

The effect of contact time on the removal of salts from the industrial brine was studied to determine how often the membrane will require to be changed. From Figure 13, the membranes show a high rejection rate during the first 5 min; the rejection rate decreases with increasing time. The continuous deposition of solutes and particles clogging the pores on the surface of the membranes is responsible for the low rejection as the contact time is increased [88]. The clogging of the pores has been confirmed by the low permeate flux (see Figure 10) compared to water flux. Several distinct mechanisms of negative rejections have been identified in studies, each characterized by different dependencies on membrane properties, feed solution composition, and operational conditions [110,111]. It has been reported that an imbalance in ion concentrations, with one or more ions present at higher levels than others, can lead to the high permeation of ions present in lower concentrations in the solution [110]. The rejection test was conducted using an industrial brine solution containing a mixture of salts at various concentrations. In this study, negative rejections were observed due to an increased concentration of salts in the membrane phase and the difference in the concentration of ions in the industrial brine solution.

### 3.4. TOC Removal Analysis

**Figure 14:**
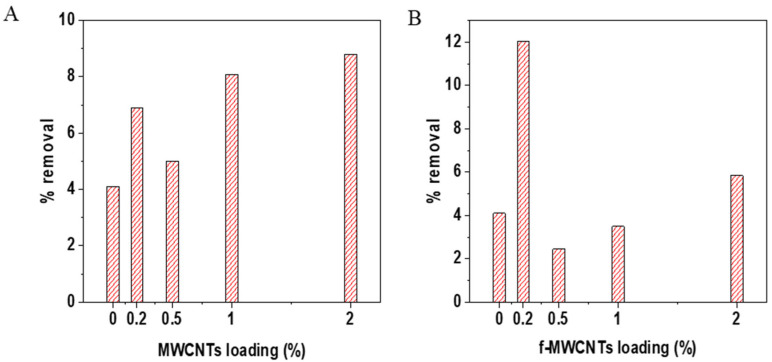
Shows effects of nanomaterials loading on the removal of TOC content in the industrial brine solution. Results for the membranes synthesized with MWCNTS are presented in (**A**), and membranes synthesized with f-MWCNTs are presented in (**B**).

Total organic carbon (TOC) in the brine solution was examined using a TOC analyser using KHP as the standard. The MWCNTs and f-MWCNTs (Figure 14) loaded membranes results showed fluctuations with different loadings. It is crucial to note that the industrial brine solution contains different organic pollutants with varying physical-chemical properties, and these can affect the organic removal performance [112].

Overall, the membranes with 0.2 wt.% f-MWCNTs had the highest removal percentage of 12% in terms of TOC. In contrast, the 2 wt.% MWCNTs were able to remove 8.5%. Thus, functionalizing the MWCNTs allows for a lower wt.% to be incorporated into the membrane for the removal of total organic carbon. However, the low 0.2 wt.% of f-MWCNTs is not ideal for the removal of key brine constituents (see Figure 12). Thus, these results may indicate that there are competitive removal processes between the organic carbon compounds present in the brine and the inorganic ions, which favour the inorganic over the organic. In addition, in terms of the TOC levels in the industrial brine tested, the initial TOC values (20 mg/L) are within permissible limits for discharge under various global regulations, but are also above acceptable concentrations for discharge in other locales. Also, when considering future trends and possible changes in policies such values may likely be considered high in the near future [113]. These factors highlight that further investigations are required using more sensitive chromatographic techniques (such as gas or liquid chromatography coupled to suitable mass spectrometry detectors) to ascertain which moieties are favoured in the removal processes, as TOC does not offer insight into the specific types of organic compounds present in the pollutant waste stream, which is important in terms of understanding the toxicity of the effluent.

The scope of this study was on the initial application of the synthesized nanocomposite membranes. Further work is needed in the investigation of usage of these membranes in systems and processes that can clean and regenerate the membranes, optimisation for long term operation, and investigating recycling and other sustainability options for these membranes.

## 4. Conclusions

Over the years, carbon nanotubes (CNTs) have attracted noteworthy attention in membrane technology due to their extraordinary electrical conductivity and mechanical strength. Functionalization of CNTs enhances their interaction with polar water molecules, improving water flux and enabling membrane operation at lower pressures. This, in turn, contributes to reduced energy consumption in water treatment processes. The purchased MWCNTs were functionalized with strong acids (sulphuric and nitric) to produce f-MWCNTs. The XRD of f-MWCNTs showed that they were oxidized without destroying the graphitic carbon, and this is validated by the presence of the (002) peak on f-MWCNTs. An increase in I_D_/I_G_ was observed on the Raman spectrum between the MWCNTs and f-MWCNTs, indicating the acid treatment was successful in the oxidation of the nanotubes. Functionalization of MWCNTs requires special attention in terms of the temperature used, the reagents used for the oxidation, and the time taken. The membrane’s hydrophilicity was improved by the addition of MWCNTs and f-MWCNTs. The high water and permeate flux obtained in this work is an indication that the membranes synthesized has the potential for application in large volumes of water. Furthermore, the use of low pressure is an added advantage of reduced energy consumption in terms of pressure. The rejection rate was improved from 6.89% on the bare membranes to 41.85% when using membranes modified with acid-treated MWCNTs. This indicates that the nanomaterials in the membranes had a significant impact on the treatment of highly saline wastewater. The scope of this study was on the initial application of the synthesized membranes, however it is important to highlight the importance of studying the re-usability and membrane cleaning properties as this is crucial for determining operational stability and sustainability. The use of dead-end cells in this study affected the rejection, due to low rotational speed causing low mass transfer, and high concentration on the membrane. An alternative that could be used is the crossflow because membranes work longer on crossflow and the feed flows parallel as opposed to the dead-end cell where the feed flows perpendicularly. Based on the rejection rate obtained in this study, it is imperative to take into consideration the pore size of the membrane in relation to the molecular weight of the salts in the brine solution to obtain higher rejection rates. In future work, we intend to investigate the integration of PDVF-based membranes with diverse nanomaterials beyond carbon-based variants. Our focus will be on comprehensively exploring their applicability in resource recovery from wastewater, aligning with the global trend towards a circular economy.

## Figures and Tables

**Table 1:** Comparison of the interspacing layer of the MWCNTs and f-MWCNTs.

Sample	2θ/°	FWHM/°	Interplanar Spacing d_(002)_/nm
MWCNTs	25.52	2.958	0.3486
f-MWCNTs	25.31	4.034	0.3515

**Table 2:** Comparison of the spectral parameters in the Raman of MWCNTs with f-MWCNTs.

Sample	D		G		
	Position	FWHM	Position	FWHM	I_D_/I_G_
MWCNTs	1449.33	22.015	1601	11.31	0.26
f-MWCNTs	1347.61	58.38	1591.07	85.48	0.85

**Table 3:** BET textural properties of MWCNTs and f-MWCNTs.

Sample	Surface Area (m^2^/g)	Pore Volume (m^3^/g)	Pore Size (nm)
MWCNTs	202.31	1.12	22.26
f-MWCNTs	244.51	1.95	31.96

**Table 4:** Contact angle measurements and WIC for the membrane and nanocomposite membranes.

Sample	Contact Angle	Water Intake Capacity (WIC)%
PVDF/PVP	85.45	75.51
PVDF/PVP/0.2% MWCNTs	79.41	79.52
PVDF/PVP/0.5% MWCNTs	77.43	73.72
PVDF/PVP/1% MWCNTs	75.76	77.06
PVDF/PVP/2% MWCNTs	72.63	76.77
PVDF/PVP/0.2% f-MWCNTs	72.57	78.39
PVDF/PVP/0.5% f-MWCNTs	71.46	73.80
PVDF/PVP/1% f-MWCNTs	70.55	78.71
PVDF/PVP/2% f-MWCNTs	68.26	78.10

**Table 5:** Comparison of rejection rates between different desalination membranes and this work.

Membrane	Salt Rejection	Pressure (kPa)	Target Source	Concentration (ppm)	Ref
PVDF/PVP/1%MWNCTs	41.8%	100	Real industrial brine	1080–14,000	This work
GO/polymer (PES)	98.5%	1000	Synthetic NaCl	2000	[106]
NMPS/PA	98.7%	1600	Synthetic NaCl	2000	[107]
TEMPO/CFNs/PA	96.2%	1500	Synthetic NaCl	2000	[108]
TFN-f-nTiO_2_/PA	98.4	1500	Synthetic NaCl	2000	[109]
	54.8	1500	Synthetic Boric acid	5	
PSf-TiO_2_	72.8%	250	Synthetic NaCl	1160	[86]

## Data Availability

The raw data supporting the conclusions of this article will be made available by the authors on request.

## Abbreviations

The following abbreviations are used in this manuscript:

PVDF	Poly (vinylidene) fluoride
PVP	Polyvinylpyrrolidone
MWCNTs	Multi-walled carbon nanotubes
f-MWCNTS	Functionalized multi-walled carbon nanotubes
p-MWCNTs	Pristine multi-walled carbon nanotubes
FTIR	Fourier Transform Infrared Spectroscopy
TEM	Transmission electron microscopy
TGA	Thermogravimetric analysis
SEM	Scanning electron microscopy
TOC	Total organic carbon
WIC	water intake capacity
KHP	Potassium hydrogen phthalate
